# Human Genetic Relevance and Potent Antitumor Activity of Heat Shock Protein 90 Inhibition in Canine Lung Adenocarcinoma Cell Lines

**DOI:** 10.1371/journal.pone.0142007

**Published:** 2015-11-11

**Authors:** Francisco Clemente-Vicario, Carlos E. Alvarez, Jennie L. Rowell, Satavisha Roy, Cheryl A. London, William C. Kisseberth, Gwendolen Lorch

**Affiliations:** 1 Department of Veterinary Clinical Sciences, College of Veterinary Medicine, The Ohio State University, Columbus, Ohio, United States of America; 2 Center for Molecular and Human Genetics, The Research Institute at Nationwide Children’s Hospital, Columbus, Ohio, United States of America; 3 College of Nursing, The Ohio State University, Columbus, Ohio, United States of America; 4 Department of Veterinary Biosciences, College of Veterinary Medicine, The Ohio State University, Columbus, Ohio, United States of America; Technische Universitaet Muenchen, GERMANY

## Abstract

**Background:**

It has been an open question how similar human and canine lung cancers are. This has major implications in availability of human treatments for dogs and in establishing translational models to test new therapies in pet dogs. The prognosis for canine advanced lung cancer is poor and new treatments are needed. Heat shock protein 90 (HSP90) is an ATPase-dependent molecular chaperone ubiquitously expressed in eukaryotic cells. HSP90 is essential for posttranslational conformational maturation and stability of client proteins including protein kinases and transcription factors, many of which are important for the proliferation and survival of cancer cells. We investigated the activity of STA-1474, a HSP90 inhibitor, in two canine lung cancer cell lines, BACA and CLAC.

**Results:**

Comparative genomic hybridization analysis of both cell lines revealed genetic relevance to human non-small cell lung cancer. STA-1474 inhibited growth and induced apoptosis of both cell lines in a dose- and time-dependent manner. The ICs_50_ after 72 h treatment with STA-1474 were 0.08 and 0.11 μM for BACA and CLAC, respectively. When grown as spheroids, the IC_50_ of STA-1474 for BACA cells was approximately two-fold higher than when grown as a monolayer (0.348 μM *vs*. 0.168 μM), whereas CLAC spheroids were relatively drug resistant. Treatment of tumor-stromal fibroblasts with STA-1474 resulted in a dose-dependent decrease in their relative cell viability with a low IC_50_ of 0.28 μM.

**Conclusions:**

Here we first established that lung adenocarcinoma in people and dogs are genetically and biochemically similar. STA1474 demonstrated biological activity in both canine lung cancer cell lines and tumor-stromal fibroblasts. As significant decreases in relative cell viability can be achieved with nanomolar concentrations of STA-1474, investigation into the clinical efficacy of this drug in canine lung cancer patients is warranted.

## Introduction

Cancer is the leading cause of mortality in dogs [1–3]. Lung cancer has an incidence of 15/100,000 dogs per year [4] and it is generally a disease of older dogs with an approximate age of 11 years old at the time of diagnosis. The most common histological subtype of canine lung cancer is adenocarcinoma, representing 74–77% of cases [5, 6]. Less than one-third of cases have localized disease, with 23% having distant metastasis, 13.5% lymph node metastasis and 34.6% diagnosed with vascular/lymphatic or intrapulmonary spread [7]. Clinical staging is an essential requirement for determining prognosis and treatment and surgical excision is the most commonly used and effective treatment modality. Dogs with solitary tumors (T1) have a median survival time after surgery of 348 days, while those that have multiple tumors of any size (T2) or tumors invading neighboring tissues (T3), have a dismal median survival time of only 58 days after a lung lobectomy [8].

The lack of good treatment options, other than surgery, has driven us to improve our understanding of the molecular basis of this cancer in dogs, in order to enhance the development of novel and rational therapies for this disease. Currently selected nonsurgical treatment options for dogs with lung cancer are based on clinical acumen, drug—treatment responses noted in other adenocarcinoma tumor subtypes, extrapolation of drug responses in people, and a few drug toxicity studies that evaluated a very limited number of dogs with advanced lung cancer. Therefore, a significant need exists to provide a rationale for treatment selection based on more robust evidence from *in vitro* and *in vivo* models of canine lung cancer [9].

Lung cancer remains the most common cause of cancer-related mortality in people. People with advanced disease are treated with medical therapy alone and have a poor prognosis with an overall five-year survival less than 15% [10]. Discovery of a spectrum of gene mutations and genomic aberrations has led to the use of targeted therapies utilizing a precision medicine approach which has been associated with often dramatic, although often short-lived, clinical benefit [11, 12]. Unfortunately, even in patients treated with first-line targeted therapy, resistance invariably develops, leaving chemotherapy as the cornerstone of subsequent therapy [13]. Pet dog translational models represent a major opportunity to better understand and treat human cancers, but lung cancer is the most common human cancer yet to be genetically dissected in dogs [14]. Because dog breeds are on the order of 100-fold more genetically simple than the human or dog populations, they are more powerful for understanding germline-genetic, environmental and gene-gene interaction risks [14]. Notably, the availability of state of the art human treatments for canine lung cancer is also dependent on this knowledge.

Heat shock protein 90 (HSP90), a molecular chaperone protein, plays a central role in regulating the folding, stability and function of many proteins that are oncogenic drivers for lung cancers. HSP90 is a highly conserved protein that folds newly synthesized proteins into their biologically active conformations preventing their aggregation. HSP90 is expressed as a 90 kDa protein with two major isoforms (HSP90α and HSP90β) and plays an essential role in maintaining cellular protein homeostasis. Co-chaperones and client proteins can modify HSP90’s mechanism of action [15–17]. Tumor cells express high levels of HSP90, which exists in highly activated complexes that are particularly susceptible to binding HSP90 inhibitors [18]. Heat-shock proteins promote tumor cell survival, growth and metastasis, even in growth-factor deprived conditions, by allowing continued protein translation and cellular proliferation [19]. These proteins provide a mechanism whereby cellular stresses experienced by cancer cells are either managed or avoided. Many oncogenes, including tyrosine kinases, transcription factors and cell-cycle regulatory proteins are clients of HSP90, and thus HSP90 is recognized as a crucial facilitator of cancer cell survival [20, 21].

Pharmacological blockade of HSP90, i.e. HSP90 inhibition, represents an alternative approach for therapeutic intervention, and has shown efficacy in both preclinical studies and clinical trials in people [22–24]. Geldamycin, a benzoquinone ansamycin antibiotic, binds to the nucleotide-binding site of the N-terminal domain of HSP90 preventing ATP binding, resulting in HSP90 inhibition. Geldamycin has poor solubility, stability and unacceptable liver toxicity in dogs at therapeutic doses therefore, analogues were developed [25]. STA-1474 is a highly soluble prodrug of ganetespib, a novel resorcinol-containing compound unrelated to geldamycin that binds in the ATP-binding domain at the N-terminus of HSP90 and acts as a potent HSP90 inhibitor. A phase I study with STA-1474 in dogs with cancer showed clinical activity with low grade gastrointestinal toxicity that was manageable with concomitant medications [26]. Inhibiting HSP90 in lung cancer is appealing as no resistance mutations have been identified, suggesting it represents a relatively stable target for drug treatment. As little is known about the efficacy of cytotoxic and small molecule inhibitors in canine lung cancer, the purpose of this study was to characterize the activity of currently used chemotherapeutic agents and the small molecule inhibitors, torceranib phosphate, crizotinib and STA-1474 and the effects of HSP90 inhibition on the mRNA expression of relevant kinases and HSP90 client proteins in two canine lung cancer cell lines. Here we show that STA1474 demonstrated biological activity in both canine lung cancer cell lines and tumor-stromal fibroblasts.

## Materials and Methods

### Cell Lines and Reagents

The BACA cell line was generously provided by Dr. Joseph J. Wakshlag, Cornell University College of Veterinary Medicine (Ithaca, NY). The BACA cell line was established from a histologically confirmed canine primary lung adenocarcinoma. Immunostaining of the cell line was positive for cytokeratin indicating epithelial origin [27]. The CLAC cell line was purchased through an approved materials transfer agreement with the Japan Health Sciences Foundations, JCRB Cell Bank (Osaka, Japan) [28]. Both cell lines were maintained in high-glucose Dulbecco modified Eagle medium (DMEM) with GlutaMax (Invitrogen, Carlsbad, CA) and supplemented with 10% heat-inactivated fetal bovine serum (FBS), and a penicillin- (100 I.U./ml) streptomycin (100 μg/ml) solution. Cells were passaged at ~90% confluence. *In vitro* experiments were performed when cells were ~90% confluent. STA-1474 was kindly provided by Synta Pharmaceuticals^®^ (Lexington, MA). Crizotinib and toceranib were provided by Zoetis^™^ (Groton, CT). Carboplatin (Teva Pharmaceuticals Ltd, Sellersville, PA), gemcitabine (Accord Healthcare, Durham, NC) and vinorelbine (Mylan Institutional, Rockford, IL) were purchased from The Ohio State University Veterinary Medical Center Pharmacy (Columbus, OH).

### Comparative Genomic Hybridization Array

We custom designed a 966,903 feature comparative genomic hybridization (CGH) array tiling the canine genome (C.E.A and J.L.R, manuscript in preparation; see also [29, 30]. The array is comprised of isothermal 60-nucleotide probes targeting all regions of unique or low copy repeats (i.e., with otherwise-unique sequence) based on the CanFam2 assembly (including the unmapped contigs annotated as chrUn). Average spacing of probes is 1.9kb for unique sequence and 1.2kb for low copy repeats. The CGH platform and probe design method is Agilent SurePrint G3 (it includes 7,113 additional Agilent control probes). DNA quality control, array hybridization and scanning were performed by Asuragen^®^ (Austin, TX) under Agilent certified conditions. The two tumor cell line samples were compared against a healthy male Labrador retriever as the reference. The reference specimen was obtained under informed owner consent and the following Ohio State University IACUC approved protocol (2010A0015-R1, Canine Specimen Collection and Banking) which covered the procedure used to obtain the sample and their subsequent use for research application. Agilent uses a linear normalization process (including dye based normalization using copy-neutral normalization probes) for their LogR values. This data was imported into Golden Helix SNP and Variation Suite, and converted from LogR10 to LogR2. Sample quality metrics were performed, including percentile based Winsorizing, derivative log ratio spread, and wave detection/correction. For segmentation, we used the univariate Optimal Copy Number Analysis Module (CNAM in Golden Helix), which uses a change-point identification algorithm. While this powerful algorithm accurately identifies changes in sequential data, it is computationally intensive. We selected 10 max segments per 10,000 bases, 20-marker minimum for a copy number call, and a max pairwise permuted *p*-value of 0.005 (with 2000 permutations per pair). CNV calls were based on a logR2 ratio threshold of -0.40/0.40 for losses and gains, respectively (Excel worksheet #1, S1 Table). Because this segmentation algorithm is more sensitive to deletion copy number changes, we used a more stringent threshold for deletions for further analysis (-0.45 for deletions and 0.4 for gains). Mean symmetric smoothing was applied to all figures.

### RNA Isolation and Reverse-Transcriptase Polymerase Chain Reaction

Total RNA was isolated from both cell lines using the Absolutely RNA Miniprep Kit (Agilent Technologies, La Jolla, CA), according to manufacturer instructions. The RNA quantity and quality was assessed with a NanoVue Spectrophotometer (GE Healthcare, Piscataway, NJ). TaqMan^®^ Reverse Transcription Kits (Applied Biosystems, Carlsbad, CA) were used to make cDNA for RT-PCR analysis of all gene transcripts. Primer sequences used for the RT-PCR reactions are listed in Table 1. Additional primer pair sequences used for RT-PCR reactions are referenced in Mariotti *et al*. [31]. The amplified cDNA products were separated according to size using gel electrophoresis. Amplicons were resolved on a 1% agarose gel to visualize the products. NADH dehydrogenase (ubiquinone) 1 alpha subcomplex, 1 (NDUFA1) was used as a housekeeping gene [32].

**Table 1:** Canine RT-PCR Primers.

Gene	Forward	Reverse	Product size (bp)
**AKT**	5'-TGCTTAAGAAGGACCCCAAGC-3'	5'-GCTGGTCCAGTTCGAGGGA-3'	**253**
**ALK**	5'-CTGTATCGGGGTGAGTCTGC-3'	5'-CAGGGCCTGGACAGGTTAAG-3'	**245**
**c-KIT**	5'-GATGGCCCCTGAGAGCATTT-3'	5'-GCCTTTTCAGGGGATCAGCA-3'	**248**
**c-MET**	5'-GAGGAATGTCCCACTGGAGC-3'	5'-TGCTGTCCCTCGACCATTTG-3'	**297**
**EGFR**	5'-TGGTCCTGGGGAATTTGGAA-3'	5'-GGTTATTGCTGAAGCGCACA-3'	**289**
**ErbB2/HER2**	5'-CCCGAGACCCACCTGGATA-3'	5'-CAGGGCGTAGTTGTCCTCAA-3'	**228**
**HSP70**	5'-AGCTGGAGCAGGTGTGTAAC-3'	5'-GGGGAAGAAGTCCTAATCCACC-3'	**146**
**HSP90AA1**	5'-ACCGAACTGGCTGAAGACAA-3'	5'-GATCACTTCCAGGCCATGCT-3'	**285**
**HSP90AB1**	5'-TGTCCGCCGTGTGTTTATCA-3'	5'-GCGCCGGTTAGTGGAATCTT-3'	**280**
**HSP90B1**	5'-TGAAACTGTTGAGGAGCCCA-3'	5'-GTGACTTCCCCTTCAGCAGT-3'	**288**
**NDUFA1**	5'-AATATTATAAATGGGAGGCGCGG-3'	5'-TAGTGAACCTGTGGATGTGCG-3'	**168**
**NKX2-1/TTF-1**	5'-GACGTGAGCAAGAACATGGC-3'	5'-CAGATTTTGACCTGCGTGGG-3'	**298**
**PDGFRα**	5'-CGACTCCGTTCTCAGTGTCT-3'	5'-CTGTTCCGCATGGTGTCCT-3'	**223**
**PDGFRβ**	5'-CCACGCCTCTGACGAGATTT-3'	5'-CTGCACAGCAGTGTACAGGA-3	**259**
**RET**	5'-AGCAGGATACACGACCGTTG-3'	5'-GTCCCGATGGACAAGCTTCA-3'	**384**

### Sequencing and Sequence Alignment

Standard PCR was used to generate high fidelity *Taq* polymerase-amplified PCR products. The resolved PCR products were extracted from the gel, purified using QIAquick PCR Purification Kit (Qiagen, Germantown, MD) and sequenced using BigDye^™^ Terminator Cycle Sequencing chemistry (Applied Biosystems, Carlsbad, CA). Automated Sanger capillary sequencing reactions for were run on a 3730 DNA Analyzer. Sequence alignments for the genes *HSP70*, *HSP90AA1*, *HSP90AB1*, *HSP90B1*, *MET*, *NDUFA1* and *NKX2-1* were made to the reference sequences NCBI: [*Canis lupus familiaris* (dog)]Gene ID: 403612, 480438, 474919, 404019, 403438, 481033, and 403940 updated on 7-Dec-2014 and 29-Jan-2015) using the ClustalW procedure in DNASTAR Software Lasergene MegAlign^®^ v.12.1 Madison, WI. All other primer sets used to generate PCR products were previously sequenced and aligned to verify the amplicons [31]

### Cell Proliferation Assay

To assess relative cell proliferation, cells were seeded in 96-well plates in 100 μl of DMEM supplemented with 10% FBS and incubated overnight. 2.5 x 10^3^ and 4 x 10^3^ cells per well were seeded for CLAC and BACA, respectively in order to achieve ~ 90% confluency. Plates were then treated with increasing concentrations of gemcitabine, vinorelbine, carboplatin, crizotinib, toceranib phosphate or STA-1474 and were evaluated after 72 h, using the CyQUANT^®^ cell proliferation assay according to the manufacturer instructions (Molecular Probes, Eugene, OR). For each drug and concentration, six wells were used. Briefly, 72 h after treatment, media was removed by gently inverting the plates and the plates were frozen at -80°C. The following day, plates were thawed at room temperature (RT) and 200 μl of CyQUANT^®^ GR dye/cell-lysis buffer was added to each well. Plates were incubated at RT for 5 min, protected from light and then fluorescence measurements were made using a plate reader (Molecular Devices, Sunnyvale, CA), with excitation at 485 nm and emission detection at 530 nm. Relative cell number was calculated as a percentage of the control wells: absorbance of sample/absorbance of DMSO treated cells x 100.All proliferation experiments were repeated three times.

Similarly, 4 x 10^3^ cells of BACA and CLAC were seeded per well and incubated overnight before a 72 h treatment with increasing concentrations of VER155008 and STA-1474. Cell proliferation was evaluated with CyQUANT^®^ cell proliferation assay as described above. The drug concentrations were selected based on predetermined half maximal concentration 50% (IC_50_) values for each drug. For the VER155008 and STA-1474 studies, fixed constant ratio drug combinations from 0.0625 to 16*X*, where *X* is the IC_50_ were evaluated using a minimum of 9 data points which were each repeated in triplicate. The combinations were evaluated for synergism, additive effects or antagonism by median-effect analysis (CompuSyn Software v. 1, Inc, Paramus, NJ) [33]. The nature of the combinatorial interactions was evaluated using the combination index (CI) method. Briefly, cytotoxic effects of the drug combination are described by the equation *f*_*a*_ */f*_*u*_ = [*D*/*D*_m_]^m^ where *f*_*a*_ is the fraction of cells affected, *f*_*u*_ is the fraction of cells not affected (1-*f*_*a*_), *D* is the dose of drug, *D*_*m*_ is the dose of drug to cause the median effect and *m* is the slope of the median-effect curve. The CI value definitions are listed in the Table 2.

**Table 2:** Combination index (CI) value ranges with verbal descriptors.

*CI*	*Description*
**<0.10**	**Very Strong Synergism**
**0.10–0.30**	**Strong Synergism**
**0.30–0.70**	**Synergism**
**0.70–0.85**	**Moderate Synergism**
**0.85–0.90**	**Slight Synergism**
**0.90–1.10**	**Nearly Additive**
**1.10–1.20**	**Slight Antagonism**
**1.20–1.45**	**Moderate Antagonism**
**1.45–3.30**	**Antagonism**
**3.30–10.0**	**Strong Antagonism**
**>10.0**	**Very Strong Antagonism**

### Detection of Apoptosis

Alexa Fluor 488 annexin V and 1 μl of PI solution (100 μg/ml) were used to stain cells for fluorescence-activated cell sorting (FACS). After 15 min of incubation at RT, 400 μl of annexin-binding buffer was added and the sample was gently mixed and kept on ice until analysis. Cells were analyzed within 30 min of staining. Caspase 3/7 activity was evaluated with the SensoLyte^®^ Homogeneous AMC Caspase 3/7 Assay Kit (AnaSpec, Fremont, CA) according to the manufacturer’s instructions. Briefly, 4,000 cells per well were seeded in 100 μl of medium and incubated overnight. The next morning, cells were treated with STA-1474 (0.05–1 μM) for 24 and 48 h. Then, 50 μl of the caspase 3/7 substrate solution was added to each well and mixed in a plate shaker for 60 min at 150 rpm and wrapped in foil to protect from direct light. Fluorescence intensity was measured using a plate reader (Molecular Devices), with excitation at 245 nm and emission detection at 442 nm.

### Protein Isolation

For the measurement of HSP90 and HSP70, client proteins and phosphorylated forms in the BACA and CLAC treated cells, cells were grown to 90% confluence in 100-mm dishes, placed on ice, rinsed with ice-cold DPBS, and lysed with 1X cell lysis buffer (#9803, Cell Signaling Technology^®^, Danvers, MA) with 1mM phenylmethanesulfonyl fluoride and protease inhibitors (Halt Protease Inhibitor Cocktail Kit; Pierce, Rockford, IL) added just before use. Cells were scraped from the dishes, and the lysates were incubated in the buffer for 15 min on ice, and centrifuged for 20 min at 16,000 x *g* at 4°C. The supernatants were collected, and protein concentration was determined by a modified Bradford method (Bio-Rad Laboratories, Inc, Hercules, CA).

### Immunoblot Analyses

For immunoblot studies, BACA and CLAC cells were seeded in triplicate at a density of 3.5 x 10^6^ cells per 100 mm plate and incubated overnight. All immunoblot analyses represent protein expression after 72 h of treatment. Cells were treated with 0.05, 0.25, 0.75, and 1 μM of toceranib phosphate and 0.005, 0.05, 0.5 and 1 μM of STA-1474 and were incubated for 72 h. For immunoblot analysis, 3X Laemmli sample buffer with 1 mM β-mercaptoethanol was added to 40 μg of protein extract (35–200 μg) at a final concentration of 33%, and the samples were heated at 100°C for 5 min. Cell protein extracts were fractioned on a precast 12% NuPAGE^®^ Novex^®^ Bis-Tris polyacrylamide Mini gels (10 cm x 10 cm) using NuPAGE^®^ MES SDS running buffer for small proteins (2–200 kDa) or NuPAGE^®^ MOPS SDS running buffer (Life Technologies, Grand Island, NY) for medium-size proteins (14–200 kDa) followed by electrophoretic transfer to nitrocellulose membranes (Pall Life Sciences, Ann Arbor, MI). The membranes were incubated overnight at 4°C with 0.2% Tween-20 in TBS and 2% bovine serum albumin with rabbit monoclonal and polyclonal antibodies. The blots were incubated with secondary anti-rabbit IgG horseradish peroxidase—linked antibody in 0.2% Tween-20 in TBS and 2% nonfat dry milk for 1 h at RT. Primary antibodies were epidermal growth factor receptor (EGFR, Santa Cruz Biotechnology, Inc., #sc-03; 1:500)[34], STAT3 (antibody #610189, BD Transduction Biosciences, Franklin Lakes, NJ, 1:2000), HSP90 (ADI-SPA-835, 1:600), HSP70/HSP72 (ADI-SPA-810, Enzo Life Sciences, Inc, Farmingdale, NY, 1:2000) insulin growth factor I (IGF-IRβ) receptor beta (#3027, 1:500), HER2 (#4290S, 1:1000), phospho-AKT (#4060P, 1:2000), mTOR (#2972S, 1:1000), phospho-mTOR (#2971S, 1:1000), MAPK (#4695P, 1:1000), phospho-MAPK (4060P, 1:1000), phospho-STAT3 (#9134P, 1:250) phospho-S6 ribosomal protein (#2211S 1:1000), S6 ribosomal protein (#2217S, 1:1000), β-actin (#4970S, 1:1000), GAPDH (#5174P, Cell Signaling Technologies^®^, Danvers, MA, 1:1000)[35]. Two wells of each pre-cast gel were loaded with markers, one for protein electrophoresis transfer from gel to membrane marker (#10748–010, BenchMark^™^ Pre-stained protein ladder, Life Technologies) and a protein standard marker (#LC5602, MagicMark^™^ XP Standard, Life Technologies).

### Spheroid and Fibroblast Proliferation Assays

Self-assembled clusters of cell colonies cultured in a microenvironment where cell-cell interactions dominate over cell-substrate interactions were generated in the form of 3-D spheroids. Monolayer cultures were plated at the same time for comparison of treatment effects. In both cases, 5,000 cells per well were seeded and allowed to grow for three days. For the spheroid growth, ultra-low attachment plates (Corning^®^ Costar^®^ Ultra-low attachment multiwall plates, Sigma-Aldrich, St. Louis, MO) were used. For the monolayer model, cells were plated in 96-well black flat-clear bottom plates (Greiner Bio-One GmbH, Frickenhausen, Germany). The wells that formed the perimeter of the 96-well plate (two outer rows) were filled with PBS to minimize edge effect. After 72 h of growth, cells were treated with DMSO or 0.005–10 μM of STA-1474 for 72 h. Then, viability was assessed with the CellTiter-Glo^®^ Luminescent Cell Viability Assay (Promega, Madison, WI) according to manufacturer instructions. Briefly, spheroids were disrupted and mixed by repeated pipetting, aspirated and transferred into a well of a black, flat-clear bottomed multiwell plate in 100 μl of media. Next, 100 μl of CellTiter-Glo^®^ reagent was added to each well. Contents were mixed for 2 min using a shaker to induce lysis and plates were incubated at RT for 10 min before luminescence was recorded using a plate reader (Molecular Devices). Finally, luminescence was normalized to the control group and ICs_50_ were calculated for each cell line and growth model.

For the fibroblast assay, 1.2 x 10^4^ cells were seeded per well in 500 μl of medium, incubated for 24 h and then treated with STA-1474 (0.001–1 μM) for 72 h. Viability was assessed with the CyQUANT^®^ Assay (Molecular Probes) according to the manufacturer instructions. Fluorescence was measured using a plate reader (Molecular Devices), with excitation at 485 nm and emission detection at 530 nm and results were normalized to the control group.

### Statistical Analyses

Experiments were performed three times and the data presented as mean values ± SD. Statistical analysis of significance was performed using the One-Way ANOVA followed by Bonferroni test. For non-normal distribution, a Kruskal-Wallis test followed by Dunns test was used. IC_50_ calculations were made using a logarithmic regression curve with Prism^®^ 5 for Mac OS X (GraphPad Software, Inc. La Jolla, CA). *P*-values <0.05 were considered statistically significant.

## Results

### Structural variation in the BACA and CLAC cell lines authenticates their relevance as a comparative oncogenomic model for human NSCLC

**Fig 1:**
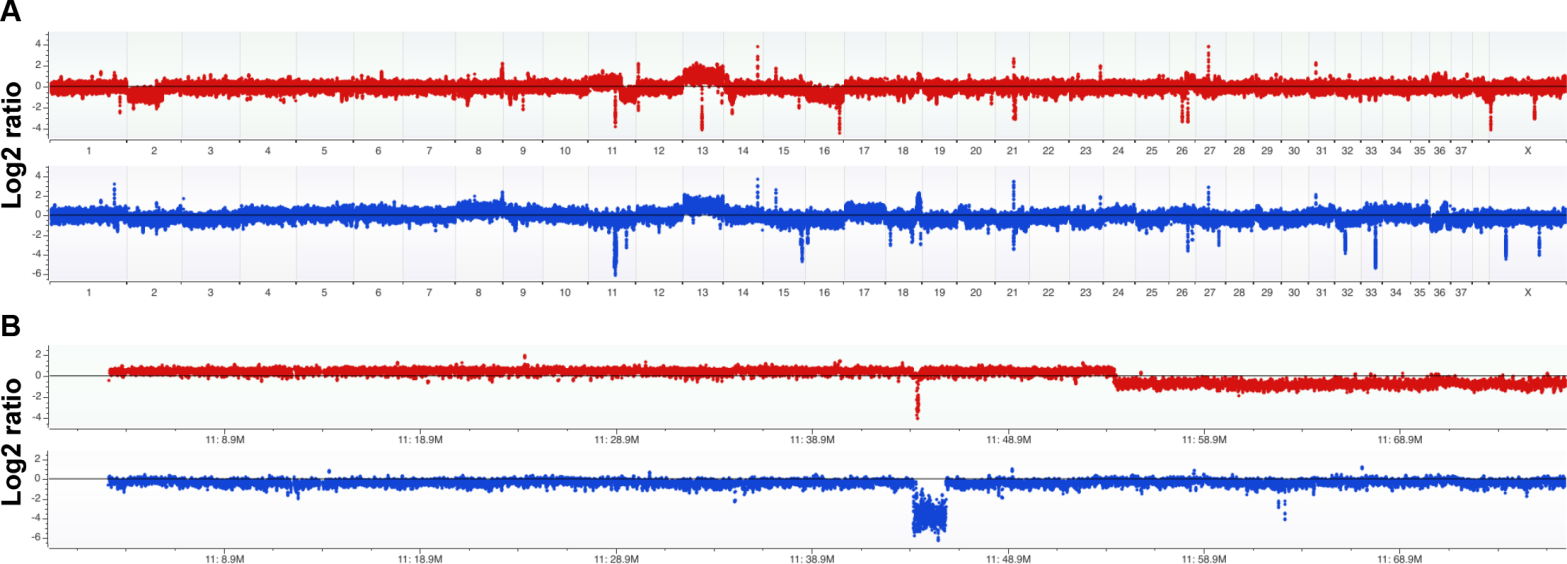
Comparative genomic hybridization analysis of canine BACA and CLAC lung adenocarcinoma cell lines. A custom 1M feature array was used to measure the DNA copy number across the genome relative to germline DNA from a normal dog. The zero line corresponds to a ratio of 1:1 between test DNA and a male reference DNA, or generally a copy number of 2. Gains are above the line and losses below. (A) Whole genome copy number analysis is shown with BACA on top (red) and CLAC on bottom (blue). Among the important findings, both cell lines have a 2-copy loss of *CDKN2A/B/B-AS1* (encoding tumor suppressors p14-p19/ARF and p16/INK4; also shown in close-up in B panels) and a 2-copy gain of chr13 (which contains the oncogene *MYC*). Other large alterations are a 1-copy loss of most of chr2 and chr16 in BACA and a 1-copy gain and loss of chr17 and chr11, respectively in CLAC. Most of all other variants shared by the two cell lines represent Copy Number Variation (CNV) in the reference DNA. (B) Copy number analysis of complete chr11 shows BACA has a 1-copy gain over 2/3rds of its length and a 1-copy loss over the remainder and CLAC has a 1-copy loss over its full length. Both lines have a relatively small 2-copy loss (focal in BACA) that overlaps only *CDKN2A/B/B-AS1* between the two cell lines.

Comparative genomic hybridization was conducted on BACA [27] and CLAC [28] to establish the genes affected by genomic alterations or Copy Number Alterations (CNAs; Fig 1A) [14, 29, 30]. Stringent thresholds (0.4 and 0.45 Log2 ratios) and high minimum-number of probes per CNA segment (20 probes) were applied. All CNAs are provided in S1 Table, segregated according to cell line and size (focal, defined as <3Mb, and large). Many large alterations affect known cancer driver genes in these cell lines. For example, chr13, which contains the *MYC* gene that is commonly amplified in human lung adenocarcinoma, has 2-copy gains in both cell lines. Both cell lines have 2-copy loss of the most commonly deleted genome segment in human lung adenocarcinoma—which contains the genes *CDKN2A/B/B-AS1* (one focal, the other larger CNA; Fig 1B). BACA has large CNA deletions of the lung adenocarcinoma tumor suppressor *PIK3R1* (<1% of human cases) [36] and the pan cancer tumor suppressor *CASP3*. CLAC has a large CNA gain including *NRAS*, a known lung adenocarcinoma driver in 0.4% of human cases [36]. CLAC also has gain of the lung adenocarcinoma oncogene *CCND1* associated with 4% of human cases.

Because the large CNAs contain very high numbers of presumptive bystander genes, it is not straight forward to evaluate all as potential oncogenic drivers. However, it is possible to study focal alterations—here arbitrarily defined as <3Mb—to implicate known cancer genes and pathways. Table 3 shows the results of Cancer Gene annotation system for Cancer Genomics (CaGe) analysis of all identified CNAs [37]. The table was also annotated by manual analysis to consider whether a gene is likely to be a tumor suppressor or oncogene [38]. Using our conservative criteria to minimize false positives, the total number of genes affected by focal alterations is 263. Of those, 129 genes were called as either cancer drivers (89 genes) or pathway genes (40). We determined that at least 28 and 13 of those genes, respectively, are gained or lost in the predicted direction to be oncogenic (i.e., gain of oncogenes and loss of tumor suppressors). Two of those genes–*CDKN2A* (Fig 1B) and *LRP1B* –are reported to be significantly mutated in lung adenocarcinoma (but the latter, called only in the earlier study, may have been due to the large size of the gene) [36]. Gene Set Enrichment Analysis (GSEA) of all focal CNA genes yielded the top match by significance as genes altered in a complex therapeutic model (GSEA gene set name Zhang_antiviral_response_to_ribavirin_dn) applied to the human lung adenocarcinoma A549 cell line; another top hit was genes down-regulated by stable expression of *SEMA3B* in the human lung adenocarcinoma cell line H1299 (*p*-values/false detection rate *q*-values of 4.5E-06/3.27E-02 and 2.99E-05/4.65E-02). While those mechanistic studies with human cell lines may not be directly relevant to the canine BACA and CLAC cell lines, this finding and the implicated driver genes mentioned indicate that the dog lines are relevant to human lung adenocarcinoma.

**Table 3:** Gene annotation summary of focal copy number alterations in BACA and CLAC.

*Annotation Category*	*Genes*
CGI Genes	*TSC2*, **CDK6**, CDKN2A*, **SMO**, CHEK2, *BCL6*, **LPP**, **NF2**, RANBP17, CRTC3
CGI (not CGC)	**ARHGDIG**, CARD8, **CTSC**, DUSP10, IRF5, MAGI2, **MAPK8IP3**, NRF1, PEX1, **RAB11FIP3**, RPS27L, TUSC3, UBE2E3, UBE2H, UBE2I, AHCYL2, CYP51A1, **KAT2B**, MAD2L1, TNFRSF1B, AXIN1, NME3, PARK2, HMGCS2, SMS, ATP6V1F, **IL1RAP**, FLNC, **SDC1**, SGOL1, CALU, RAB5A, **APOB**, **SSTR5**, CACNA1H, IMPDH1, IQGAP1, **CLDN16**, LEP, CCL20, **CDKN2B**, MPG, NTHL1, CLDN1, DMD, RHOB, AP1B1, **TRIO**, **B3GALNT1**, **GALNT3**, *ARHGAP27*, ATAD3B, BAIAP3, CCDC136, **DNAH5**, DYNLRB2, FAM190A, **GFER**, KRIT1, LRP1B*, LRRC4, MRPL28, MSLN, MSR1, **NLRP5**, OCA2, OSR1, **PKD1**, PKP4, PNN, RASL10A, RHBDD3, RHBDF1, **SCN9A**, SMC4, **SOX8**, SSU72, **TP63**, **ZFHX3**
CGC & CGI Pathway	ARHGAP15, ARHGAP24, **DECR2**, DIAPH2, GDF7, GNPTG, HERC2, LAPTM4A, NEFH, **PIWIL2**, SEC23A, SLC9A3R2, SPHKAP, STUB1, IGFALS, **PPM1L**, **PIGQ**, **IL12A**, RPL38, RPL3L, GEMIN2, **MASP1**, **GNG13**, OPN1SW, **PPP3CC**, POLR3D, CTNNA3, NDUFB10
CGI (not CGC) Pathway	SLC39A14, HAGHL, SLC19A3, KYNU, **OR14C36**, **OR14I1**, **OR2G6**, OR2T11, **OR2T27**, **OR2T6**, OR51F1, SLC39A14

Cancer Gene Census (CGC), Wellcome Trust Sanger Institute (http://cancer.sanger.ac.uk/census/; Futreal *et al*. PMID: 14993899); Cancer Gene Index (CGI), National Cancer Institute (https://wiki.nci.nih.gov/display/cageneindex/Cancer+Gene+Index+End+User+Documentation); bold, genes in the correct direction of gain/loss to be oncogenic or tumor suppressive (according to analysis of pan cancer sequence mutation profile by Davoli *et al*. PMID: 24183448); italics and underline, genes that can be either oncogenic or tumor suppressive (according to Davoli *et al*. PMID: 24183448); asterisk, genes among 26 significantly mutated genes in human lung adenocarcinoma (FDR < 0.1; according to Ding *et al*. PMID: 18948947)

### cDNA transcripts are present for NKX2-1, HSP70 and HSP90 isoforms, and HSP90 client proteins

**Fig 2:**
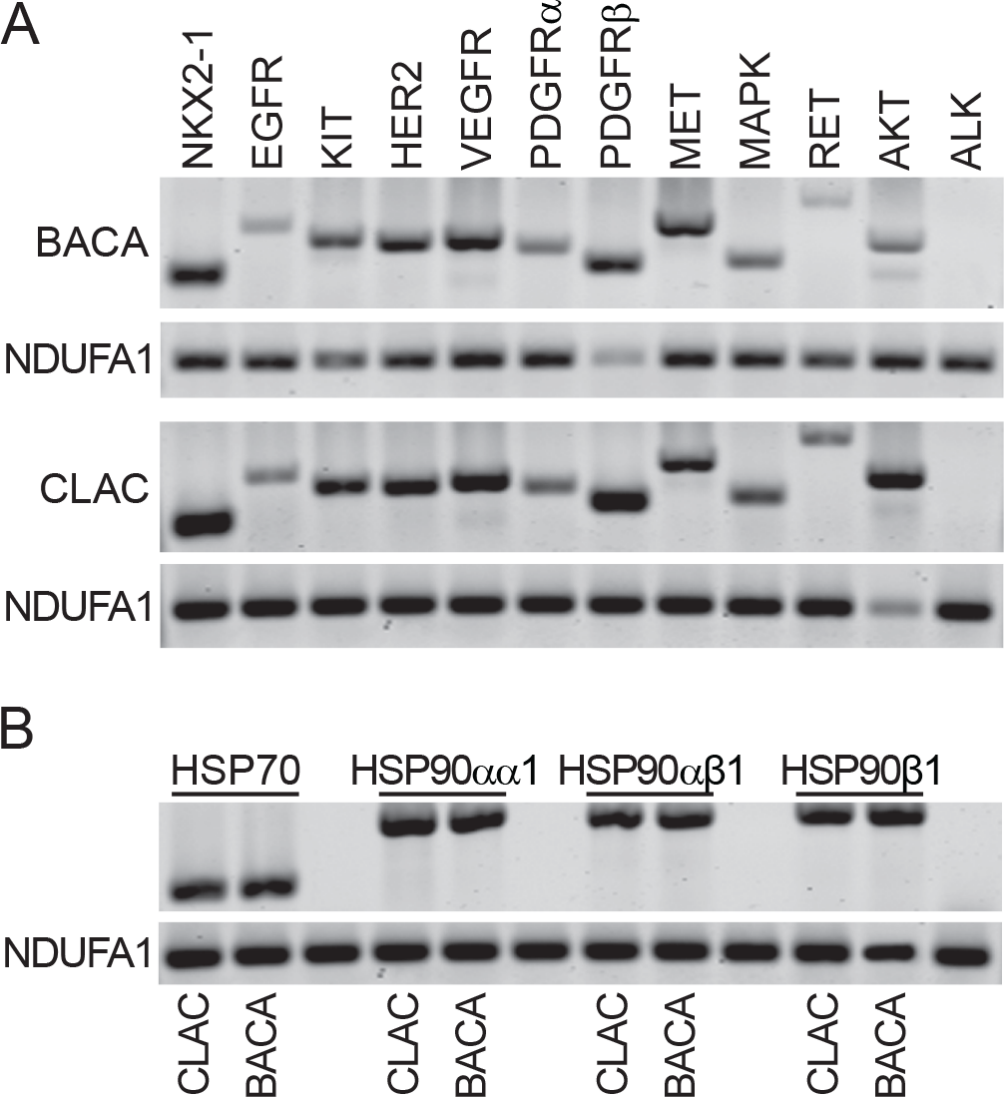
Characterization of BACA and CLAC canine lung cancer lines by RT-PCR. (A) Reverse transcriptase cDNA transcripts of NKX2-1 (lung cancer cell marker) and HSP90 client proteins in BACA and CLAC cell lines. NDUFA1 serves as a loading control. (B) Reverse transcriptase cDNA transcripts of HSP70 and three HSP90 isoforms in BACA and CLAC cell lines. NDUFA1 serves as a loading control.

Reverse transcriptase-polymerase chain reaction (RT-PCR) was used to identify the presence of mRNA for NKX2-1, also known as TTF-1, a marker of lung differentiation, and eleven receptor and cytoplasmic tyrosine and serine/threonine kinases, in the BACA and CLAC canine lung cancer lines. Both cell lines expressed NKX2-1 (Fig 2A). Ten of eleven HSP90 client kinase cDNA transcripts investigated for expression were present in both cell lines. EGFR, c-Kit, HER2, VEGFR, PDGFRα/β, c-MET, MAPK, c-RET, AKT-2 were present and transcripts for ALK were absent (Fig 2A). Both cell lines had transcripts of HSP70 and the HSP90 isoforms (Fig 2B).

### STA-1474 inhibits BACA and CLAC cell line proliferation

**Fig 3:**
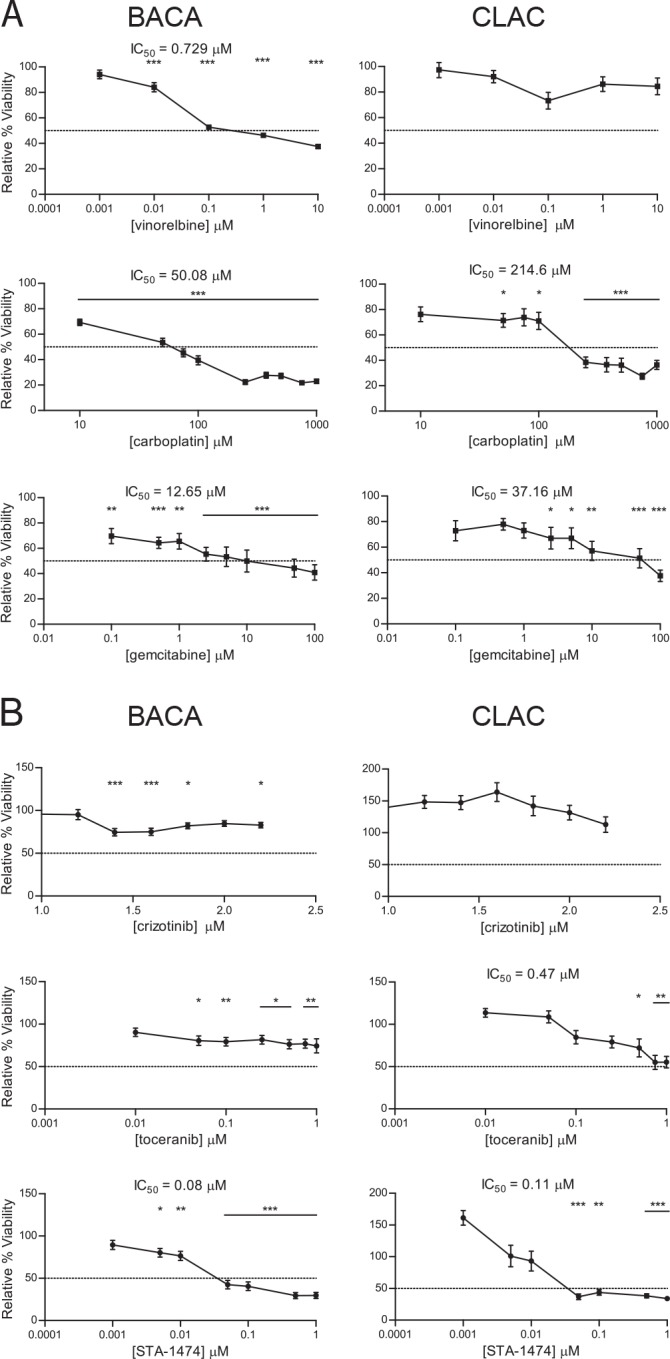
Viability assays and half-maximal inhibitory concentrations (ICs_50_) for canine lung cancer cell lines. Cells were treated with increasing concentrations cytotoxic drugs (A) or small molecules inhibitors (B) and proliferation was evaluated after 72 h. Treatment effects were normalized to the drug vehicle-treated control group. Each graph shows mean±SEM. ICs_50_ were calculated for each experiment. The dotted line in the y-axis represents the 50% relative viability. *, *p*<0.05; **, *p*<0.01; ***, *p*<0.001.

Cell viability was assessed after 72 h of treatment with increasing concentrations of drugs commonly used to treat lung cancer in humans and dogs (Fig 3A and 3B). IC_50_ values were determined for each drug in both cell lines. Effects on cell proliferation were generally dose- and cell line-dependent. With respect to currently used cytotoxic chemotherapeutics, treatment of the BACA line with vinorelbine achieved the lowest IC_50_ (0.729 μM). In contrast, when the CLAC line was treated with increasing concentrations of vinorelbine for 72 h, an IC_50_ was never reached, i.e. this cell line was relatively resistant (Fig 3A). Treatment of the CLAC line with carboplatin did not result in a dose-dependent decrease in cell viability until a concentration of 100 μM was reached. Increasing doses of carboplatin did result in decreasing BACA cell viability. Gemcitabine ICs_50_ for the BACA and CLAC lines were lower than those achieved with carboplatin. Statistically significant decreases in BACA cell viability at 72 h were present for all concentrations of gemcitabine used. The IC_50_ for gemcitabine-treated CLAC cells was approximately three times greater than that of BACA. Overall, BACA cell viability was more sensitive to these cytotoxic drugs as indicated by the lower ICs_50_ compared to the CLAC line.

Of the three different small molecule inhibitors evaluated, STA-1474, the HSP90 inhibitor, achieved the greatest inhibition of cell viability and had the lowest ICs_50_ after 72 h of drug exposure (Fig 3B). BACA cell viability was minimally affected by torceranib phosphate treatment. A concentration-dependent decrease in CLAC viability was found with increasing concentrations of toceranib resulting in a lower IC_50_ (0.47μM) when compared to BACA. Crizotinib treatment of the CLAC cell line did not produce a statistically significant decrease in viability which resulted in an unachievable IC_50_. Although critzotinib treatment of the BACA cell line produced a significant (***p<0.001) decrease in viability at both the 1.4 μM and 1.6 μM concentrations, the cell viability was higher than 50% with the doses used.

### STA-1474 promotes apoptosis in a time- and dose-dependent manner

**Fig 4:**
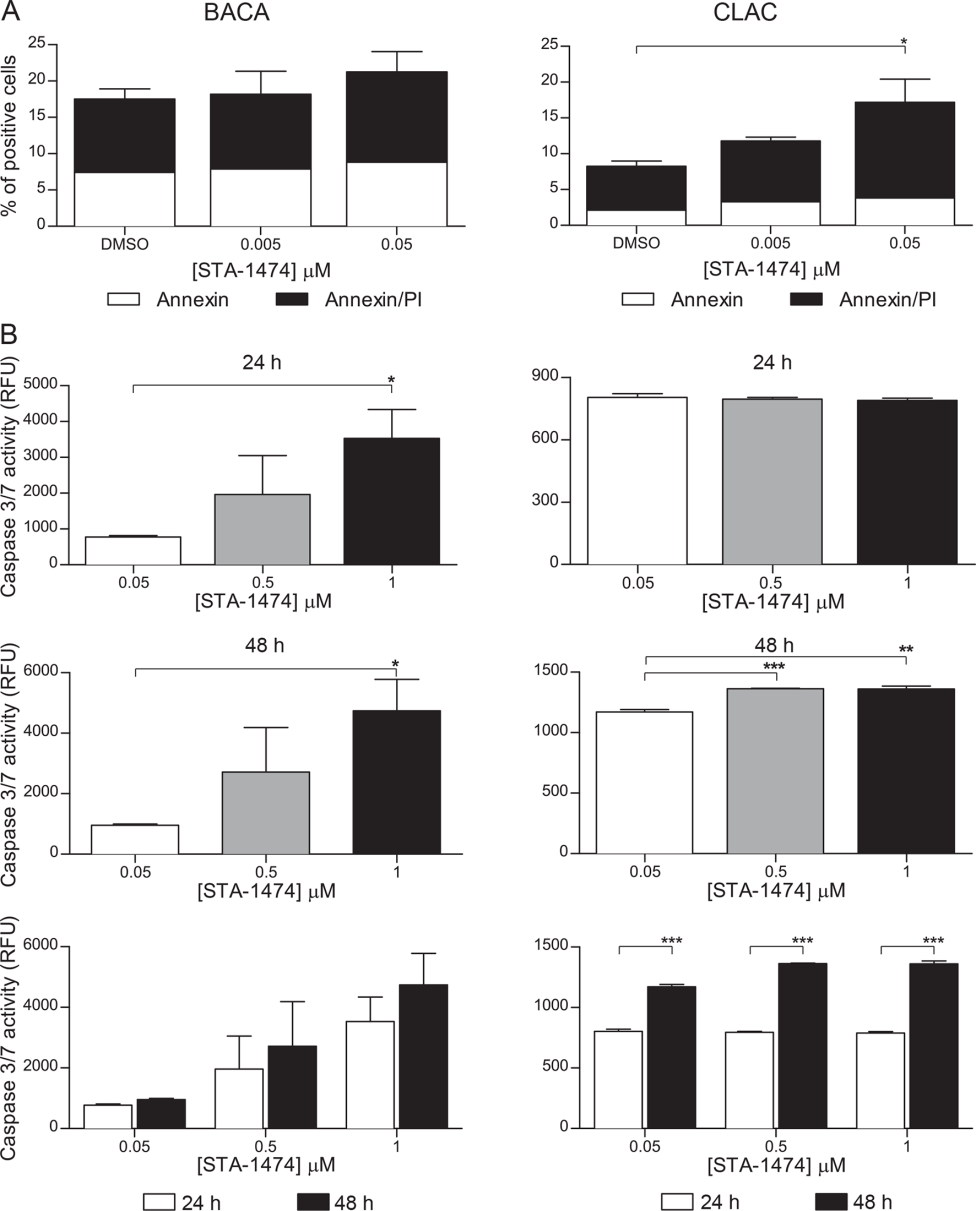
Evaluation of apoptosis in canine lung cancer cell lines treated with STA-1474. Apoptosis was assessed by annexin V/PI staining flow cytometry and detection of caspase 3/7 enzymatic activity. (A) Cells were treated with dimethyl sulfoxyide (DMSO, control) or 0.005–1 μM of STA-1474 for 24 h. Staining with annexin V and the vital dye, propidium iodide (PI), were used to evaluate early and late apoptosis. Cells that are considered viable are both annexin V and PI negative, while cells that are in early apoptosis are annexin V positive and PI negative, and cells that are in late apoptosis or already dead are both annexin V and PI positive. (B) Both cell lines were treated as above and evaluated for executioner caspase-mediated apoptosis. Activated caspases 3 and 7 were assessed 24 and 48 h after treatment. Experiments were performed in triplicate and repeated three times. Each graph shows mean ± SEM. *, *p*<0.05; **, *p*<0.01; ***, *p*<0.001.

To determine if the growth inhibitory effect of STA-1474 on both cell lines was associated with apoptosis, cell lines were treated with increasing concentrations of STA-1474 (0.005–0.05 μM) for 24 h and evaluated for annexin V and propidium iodide (PI) staining. Although incubation of the BACA line with 0.05 μM STA-1474 for 24 h increased the proportion of cells undergoing apoptosis (represented by both annexin V and annexin V & PI positivity), this was not statistically significant (Fig 4A). In contrast, 24 h exposure of the CLAC line with 0.05 μM STA-1474 resulted in a significant (*p* <0.05) increase in the proportion of apoptotic cells. Characterization of the identified apoptotic response was evaluated further by quantifying the executioner caspase activity of caspases 3 and 7 after 24 and 48 h exposures to vehicle (DMSO) or increasing concentrations of STA-1474 (0.05–1 μM). Twenty-four and 48 h treatments of the BACA line with STA-1474 resulted in a significant dose-dependent increase in caspase activity (Fig 4B, middle left panels). A dose-dependent significant increase in caspase 3/7 activity was seen in the CLAC line after 48 h of drug exposure (Fig 4B, middle right panels). The relative increase in caspase3/7 increased significantly (*p*<0.001) between 24 and 48 h in the CLAC line but not in the BACA line (Fig 4B, lower panels).

### STA-1474 decreases expression of signal transduction proteins and up-regulates HSP70 expression in canine lung cancer cells in a dose-dependent manner

**Fig 5:**
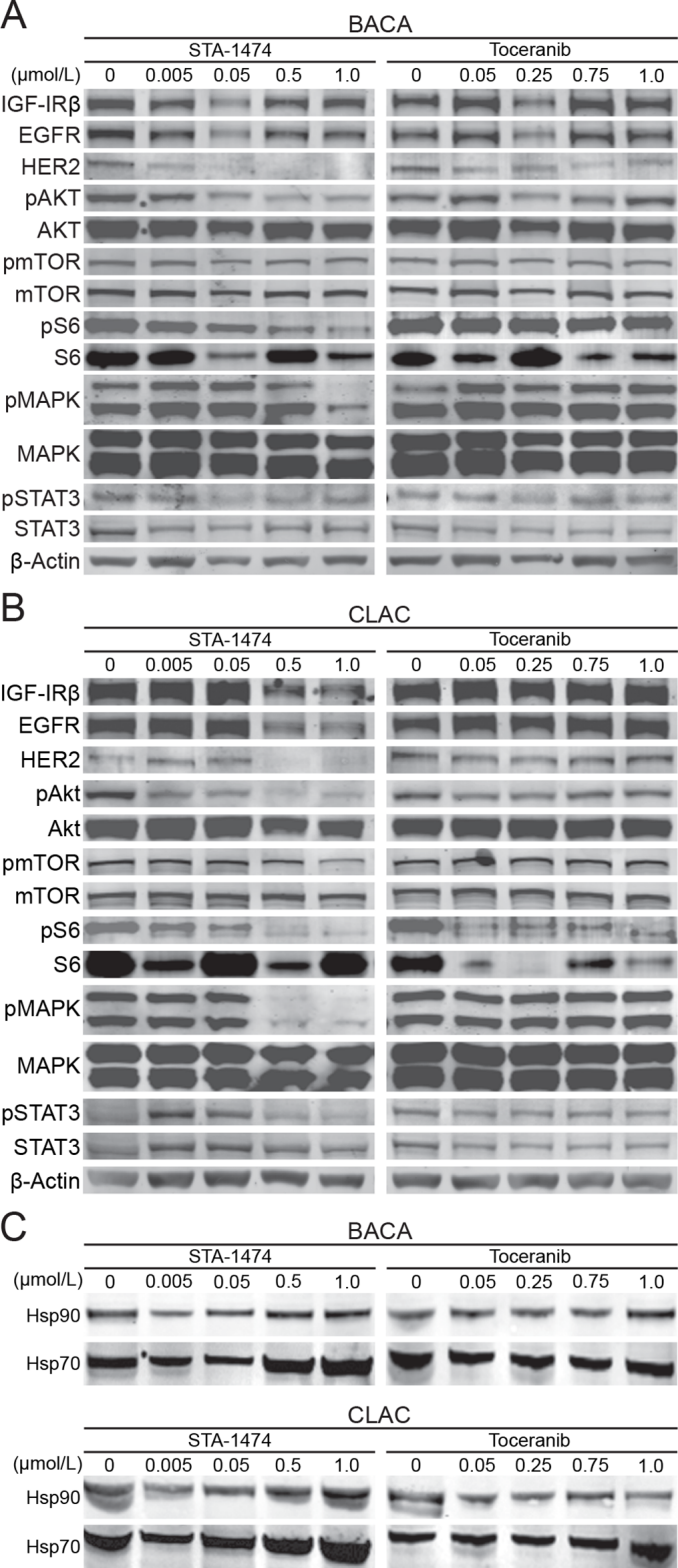
Protein expression of HSP90-regulated proteins, HSP70 and HSP90 in canine lung cancer cell lines after treatment with small molecule inhibitors. A set of three plates for each cell line was used for evaluation of total and phosphoproteins of downstream signaling pathways (A) Representative immunoblots of HSP90 client protein expression from BACA whole cell protein lysates after treatment with STA-1474 or toceranib phosphate. (B) Representative immunoblots of HSP90 client protein expression from CLAC whole cell protein lysates after treatment with STA-1474 or toceranib phosphate. Controls were cell lines treated with the drug solvent, DMSO, as represented by the 0 concentration. Evaluation of phosphoprotein forms of the proteins are indicated by “p”. Drug concentrations are μmol/L. The β-actin Western blots serve as loading controls. (C) Immunobloting from whole cell protein lysates of HSP70 and HSP90 of BACA and CLAC lines treated with DMSO (control), STA-1474 and toceranib phosphate.

To further characterize the *in vitro* activity of STA-1474 in comparison to torceranib phosphate, we assessed the ability of these compounds to deplete critical client proteins of HSP90 and multiple receptor tyrosine kinase targets of torceranib. We also evaluated the ability of these compounds to extinguish downstream signaling of the PI3K/mTOR/S6 and RAF/MEK/ERK pathways and the transcription factor, STAT3. Treatment of the BACA cell line with biologically relevant concentrations of STA-1474 induced client-protein depletion of HER2 (starting at 0.05 μmol/L) and STAT3 (at 1.0 μM). Degradation of the phosphorylated forms of the downstream signaling proteins, pAKT, pMAPK, pS6, and transcription factor, pSTAT3, occurred after exposure to 1.0 μM in the BACA cell line (Fig 5A); whereas in the CLAC cell line, decreased phosphorylated forms of the same proteins occurred at just 0.5 μM (Fig 5B). Interestingly, protein levels of pmTOR were unaffected by exposure to biologically achievable doses of STA-1471 in the BACA line and only slightly affected the CLAC line. Toceranib treatment of the BACA cell line resulted in a slight decrease in HER2. Degradation of all other proteins and phosphorylated forms were unaffected at exposures <10 μmol/L (Fig 5A and 5B).

As inhibition of HSP90 typically leads to the increased expression of other HSP family members that can be used as surrogates for HSP90 inhibition, we evaluated the effect of HSP90 abrogation on HSP70 expression. Treatment of both cell lines with increasing concentrations of STA-1474 (0.005–1.0 μM) for 48 h resulted in an increase in HSP70 expression at 0.5 and 1.0 μM exposures (Fig 5C, left panels). As expected, treatment of both cell lines with increasing concentration of toceranib did not increase HSP70 expression (Fig 5C, right panels).

### Synergistic cytotoxicity from dual inhibition of HSP70 and HSP90 is dose and cell-line dependent

**Fig 6:**
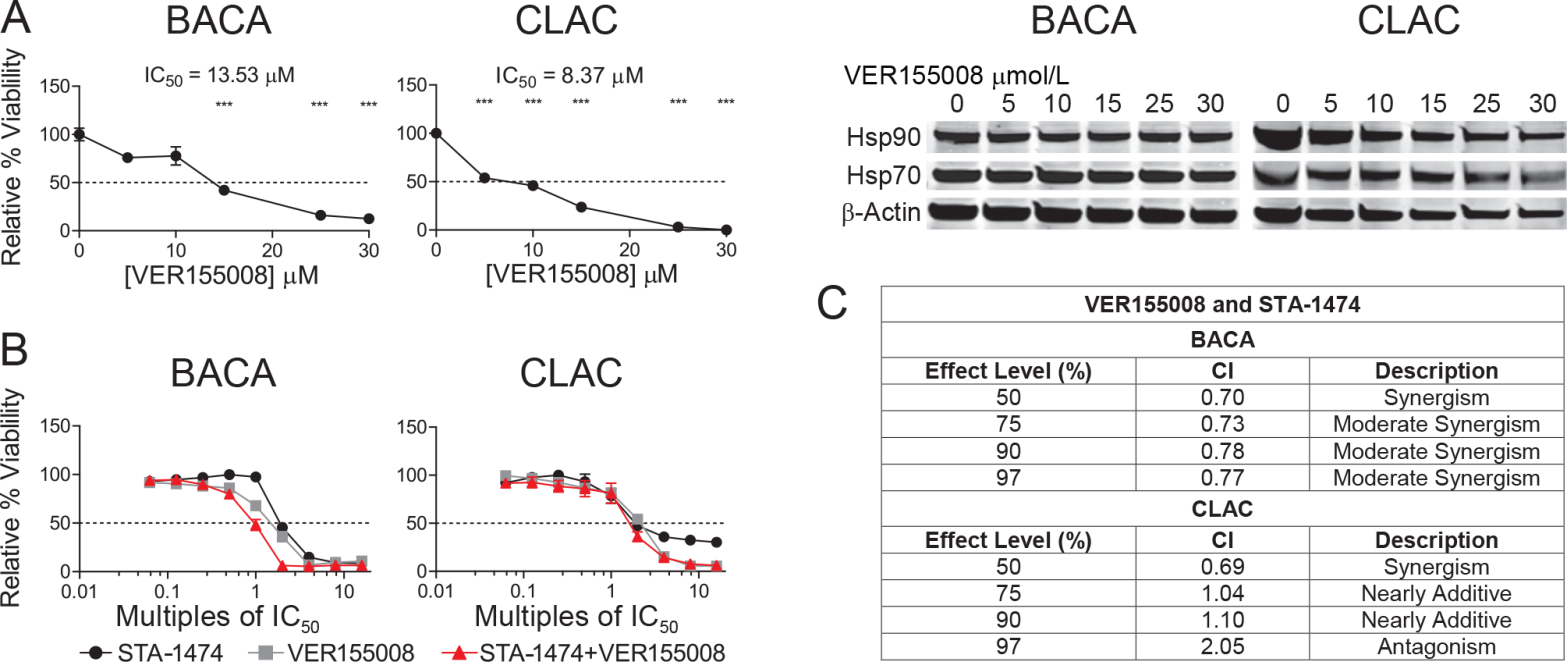
Relative cell viability assays, half-maximal inhibitory concentrations (ICs_50_) after treatment with an HSP70 inhibitor (VER155008) or a combination of STA-1474 and VER155008. (A) Cells were treated with HSP70 inhibitor, (VER155008) for 72 h. The relative viability and ICs_50_ were determined for both cell lines after treatment. (B) BACA or CLAC cells were plated in DMEM media for 24 h and then treated for 72 h with VER155008 only (gray squares), STA-1474 only (black circles) or a combination of VER155008 and STA-1474 concentrations (red triangles) ranging from 0.0625X to 16X their IC_50_ concentrations. Each graph shows mean ± SEM. Each group was compared to the DMSO control. The dotted line in the y-axis represents the 50% relative viability. *, *p*<0.05; **, *p*<0.01; ***, *p*<0.001. (C) Multi-drug combination dose-effect analysis for the doublet combination of VER155008 and STA-1474 on BACA and CLAC cell lines as measured by the combination index (CI). The CI value definitions are represented in the description column.

The binding of a client protein to HSP90 requires the cooperation of HSP90 with other chaperone proteins, HSP70 and HSP40. As induction of HSP70 was present in both cell lines after 72 h treatment with STA-1474, we sought to determine if synergistic cytotoxicity would occur when cells were treated with the combination of a HSP90 inhibitor and a HSP70 inhibitor. Cell line viability was assessed after 72 h of treatment with increasing doses of the HSP70 inhibitor, VER155008 (0–30 μM). There was a dose-dependent reduction in viability of both cell lines after treatment with VER155008 without a change in the expression of HSP70 or HSP90 in either cell line (Fig 6A). Treatment of BACA and CLAC cells for 72 h with a combination of VER155008 and STA-1474 inhibitors in concentrations that were constant ratios of multiples of the ICs_50_ reduced relative cell viability in a dose-dependent manner (Fig 6B). Dose-effect and associated CI values for the drug combination treatment (VER155008/STA-1474) at constant ratios are presented in Fig 6C. The model is most accurate at the *f*_a_ = 0.50, the point that the drugs affect 50% of the cells. BACA cells treated with VER155008/STA-1474 combinations were synergistic at *f*_*a*_ = 0.50, and with increasing two-fold IC_50_ concentration combinations, the CI values increased to produce moderate synergism at effect levels > 50%. The dose reduction index (DRI) was calculated and is a measure of how many folds the dose of each drug in a synergistic combination may be reduced at a given effect level when compared with the doses of each drug alone. The BACA line first showed evidence of synergism to the drug combination at *f*_*a*_ = 0.50 with a CI of 0.70. The DRI at *f*_*a*_ = 0.50 was 1.16 and 3.90 fold for VER155008 and STA-1474, respectively. The VER155008/STA-1474 treatment in the CLAC line provided synergism at the *f*_a_ = 0.50, and progressed to antagonistic at effect levels > 90%. The DRI at *f*_a_ = 0.50 was 1.5 and 10.5 fold for VER155008 and STA-1474, respectively. Unfortunately, the VER155008 concentration needed to decrease cell viability was too high to take advantage of the drug combination in terms of obtaining the desirable therapeutic effect by administering low doses of the drugs in combination. These results demonstrate that VER155008/STA-1474 combination has a limited dose range in which synergism is obtained and the concentrations are cell line dependent.

BACA and CLAC cells were plated in DMEM media and were exposed to the compounds for 72 h at constant fixed ratios of [75:1](VER155008:STA-1474 CLAC) and [169:1](VER155008:STA-1474 BACA) and relative cell viability assessed. The resulting CI values are shown for effect level. For example, the doses of these two drugs needed to achieve a 97% decrease in BACA relative viability gives a CI value that would indicate moderate synergism which provides a drug reduction index score indicative of a four-fold decrease of VER155008 and two-fold decrease of STA-1474.

### Relative cell viability after treatment with STA-1474 differs in monolayer cultures *vs*. tumor spheroids and STA-1474 decreases tumor-stromal fibroblast viability

**Fig 7:**
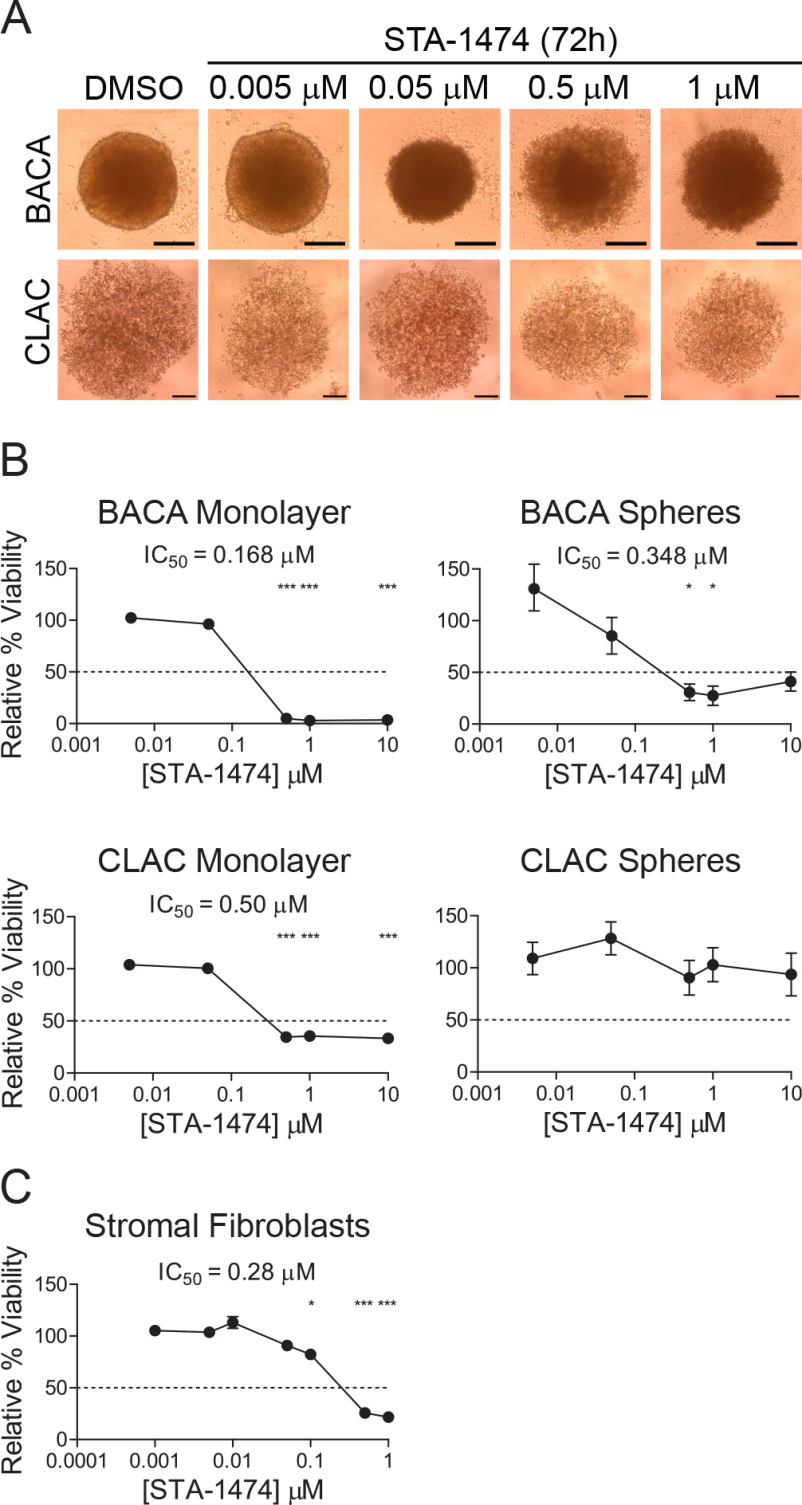
Viability and half-maximal inhibitory concentration (ICs_50_) of STA-1474 treated canine lung cancer cell-line derived tumor spheroids, monolayers and tumor-stromal fibroblasts. (A) Tumor spheroids derived from both cell lines were allowed to form for 72 h after seeding cells in the ultra-low attachment wells. Images taken of one field of view/well on an inverted microscope at 40X magnification, scale bar represents 200 μM (B) Immediately after formation, tumor spheroids were treated with DMSO or increasing concentrations of STA-1474 for 72 h. Tumor spheroids and monolayers from each cell line were grown and treated identically. Cultured cells were treated with STA-1474 for an additional 72 h and ICs_50_ were calculated. (C) Tumor-stromal fibroblasts were seeded, allowed to form a monolayer for 24 h then incubated with STA-1474 for 72 h and viability determined. Experiments were performed in four replicates and repeated twice. Each graph shows mean ± SEM and each group was compared to DMSO. The dotted line in the y-axis represents the 50% relative viability. *, *p*<0.05; **, *p*<0.01; ***, *p*<0.001.

To determine the efficacy of STA-1474 on canine lung cancer cells in different model systems, we evaluated the STA-1474 ICs_50_ for cells grown as monolayers or as three-dimensional tumor spheroids. Both BACA and CLAC cells formed tumor spheroids when grown for three days in ultra-low attachment well plates (Fig 7A). BACA cells formed compact, tight spheroids, whereas CLAC cells formed loose spheroids. Different STA-1474 ICs_50_ were obtained depending on the model used. Monolayer cultures were seeded and then allowed to grow for 72 h before treatment to mimic the growth period needed for the formation of spheroids. BACA cells grown as a monolayer, demonstrated a dose-dependent decrease in viability with an IC_50_ of 0.168 μM and <5% of cells viable at drug concentrations of 0.5 μM or higher. When grown as spheres, BACA cells were less sensitive to STA-1474 drug treatment, resulting in a doubling of the IC_50_ (0.348 μM) compared to monolayer cultures. CLAC cells grown as a monolayer had a dose-dependent decrease in viability that was significant at doses of 0.5 μM or higher. CLAC spheroid cultures were resistant to all drug treatment concentrations investigated (up to 10 μM; Fig 7B). We also evaluated the relative cell viability of tumor-stromal fibroblasts after 72 h of treatment with STA-1474. There was a dose-dependent decrease in cell viability that was significant with concentrations of ≥ 0.1 μM. The determined IC_50_ was higher than for both canine lung cancer cells lines (Fig 7C *vs*. Fig 3B).

## Discussion

The prognosis for dogs with advanced lung cancer remains poor and new treatment options are needed. The molecular characterization of canine lung cancer is limited compared to human lung cancer. In this study we wanted to investigate in canine lung cancer cells the activity of some of the molecularly targeted therapies used to treat lung cancer in people, as well as investigate other chemotherapy drugs used to treat lung cancer in dogs. The biologic activity of these different chemotherapies and small molecule inhibitors on canine lung cancer cell lines has not been reported previously and could provide guidance and insight into the choice of drugs for treating dogs with lung cancer.

Recent reports of HSP90 inhibitor activity in human non-small cell lung cancer (NSCLC) phase IIb/III clinical trials have provided compelling rationale for investigating the feasibility of using HSP90 inhibitors for treatment of lung cancer in dogs [24]. HSP90 is a highly conserved protein that folds newly synthesized proteins into their biologically active conformations preventing aggregation. HSP90 also maintains cellular protein homeostasis by acting as a molecular chaperone with its action modulated by co-chaperones and client proteins [15–17]. As HSP90 regulates multiple signaling cascades, the effects of pharmacological blockade of HSP90 should interfere with a variety of client proteins and biochemical pathways. Given that we know very little about the current crucial signaling pathways for canine lung tumor viability and considering we could target multiple signaling proteins with HSP90 inhibition, this prompted us to evaluate the pro-drug, STA-1474, in these canine lung cancer cell lines. Our initial experiments were designed to characterize the suitability of our cell lines to serve as appropriate *in vitro* models that would potentially respond to therapeutic intervention by inhibition of selected targets.

Somatic mutations are the predominant mechanism that gives rise to cancer. The average cancer cell has approximately four sequence mutations of oncogenes (mean 1) and tumor suppressors (3), 11 very large CNAs involving whole chromosomes (2 gain, 2 loss) or chromosome arms (3 gain, 5 loss), and 23 focal CNAs (11 gains, 12 losses)[38]. Here we have determined the CNAs for BACA and CLAC using high resolution array CGH. The most striking findings were 2-copy gains of chr13, which contains *MYC*, and 2-copy loss of a small part of chr11, for which the overlapping segment between the two cell lines includes only *CDKN2A/B/B-AS1*. These are among the most common CNAs seen in human lung adenocarcinoma [36], with *CDKN2A* being involved in 43% of cases. Notably *CDKN2A* encodes ARF, which directly interacts with overexpressed MYC protein to block its transformation and proliferation activities [39]. Other large CNA genes gained or lost in the correct direction to drive cancer are *PIK3R1* and *CASP3* in BACA, and *NRAS* and *CCND1* in CLAC [36]. Table 2 shows the many cancer driver and pathway genes affected in the cell lines by focal CNAs, including *TSC2*, *NF2*, *BCL6*, *CHEK2*, *CDK6*, *KAT2B*, *PKD1* and *TP63*. Among the mechanistic insights, the CNAs suggest relevance for HSP90 inhibition that would be expected to have therapeutic effects through the PI3K and MAPK pathways (e.g., *PIK3R1*, *TSC2*, *BCL6*, *NF2*, *PKD1*, and *NRAS*). Other pathways that are likely to be affected according to our findings are cell cycle progression (e.g., *CDKN2A* and *CCND1*) and apoptosis (*CASP3*). The array CGH data thus support both lung adenocarcinoma and pan cancer relevance. Additionally, the GSEA analysis of these two canine cell lines strongly implicates human lung adenocarcinoma among all other cancer data. These facts formally demonstrate that BACA and CLAC can serve as comparative oncogenomic models for development of drug treatments to mammalian lung adenocarcinomas in which a set of common driver mechanisms are present.

Once we confirmed that the cell lines were appropriate *in vitro models* to evaluate candidate drugs that have been effective in controlling human NSCLC, it was important to establish that these canine lung cancer cell lines expressed HSP90 isoforms, HSP70, and various receptor tyrosine kinases and downstream kinases, relevant to lung cancer in humans. These included EGFR, c-Kit, HER2, VEGFR2, PDGFRα, PDGFRβ, c-Met, MAPK, c-Ret and Akt. The cell lines used for this work were from primary canine lung tumors and of pulmonary cell differentiation as the presence of cDNA transcripts for TTF-1, a nuclear protein [40]. TTF-1, also known as NKX2-1, is a homebox-containing transcription factor essential for the development of the lung, and its use as a marker of lung adenocarcinoma, has been recommended by the newer classifications of human NSCLC expressed in follicular cells of the thyroid gland and pneumocytes was present. TTF-1 has a specificity of 100% and sensitivity of 85% in canine primary lung cancer [10, 11, 41]. Both cell lines had cDNA transcripts for HSP70, HSP90, and all the client proteins and other kinases investigated except ALK.

When complete surgical resection of a primary lung tumor is not possible in canine patients, treatment with cytotoxic chemotherapy may be considered in an attempted to slow the progression of the disease. Interestingly, the cytotoxic drugs used in this study had limited to no apparent effect on cell viability *in vitro*, or only had effects at drug concentrations not thought to be biologically relevant, or achievable, *in vivo*. For example, the IC_50_ of vinorelbine for BACA was 0.72 μM, which is 4- to 10-fold higher than in human NSCLC [42] and the CLAC cell line was drug-resistant. The responsiveness of our cell lines is consistent with what has been reported in the clinical setting, where only two out of seven dogs with macroscopic bronchoalveolar carcinoma had a partial response to vinorelbine treatment [43]. The obtained CLAC IC_50_ dose for carboplatin is not tolerable in dogs. The tolerated carboplatin IC_50_ has been extrapolated from pharmacokinetic data performed in laboratory beagle dogs using C_max_ at the recommended dose of 300 mg/m^2^ given as an IV bolus [44]. An estimated maximum tolerated dose of carboplatin is 250 μM [44]. Likewise, a previous pharmacokinetic study evaluating gemcitabine in dogs found the maximum tolerated dose of 22 mg/kg resulted in a C_max_ of 20–30 μg/mL which is equivalent to a molarity of 67 to 100 μM [45]. When the carboplatin ICs_50_ for the lung cancer cell lines (50 and 214 μM for BACA and CLAC, respectively) is compared to ICs_50_ for other cancer cell lines of canine origin, they are higher than then those for canine mammary gland tumors or canine melanoma (30.5 μM and 6.1 μM, respectively) [46, 47]. The gemcitabine IC_50_ differed between the cell lines, with the CLAC IC_50_ being three-fold higher than BACA IC_50_. Our IC_50_ findings for gemcitabine are similar to the ICs_50_ reported for canine osteosarcoma cell lines, which ranged from 5.7 to 15.3 μM for the cell lines that had dose-dependent decreases in cell proliferation [48].

We evaluated the prodrug STA-1474 in lieu of ganetespib because of its greater solubility in water which facilitates its use in the dog without untoward side effects. A phase I study evaluated STA-1474 in dogs with solid tumors and reported measurable objective responses for malignant mast cell disease, osteosarcoma, melanoma and thyroid carcinoma [26]. Based on the favorable evidence of STA-1474 displaying potent activity against both canine lung cancer cell lines, we further investigated the effects of HSP90 inhibition in canine lung cancer. STA-1474 decreased cell viability at 72 h, induced apoptosis and promoted activation of caspase 3/7 in a dose- and time-dependent manner in both cell lines. Apoptosis was detected 24 h after treatment and caspase 3/7 activity continued to increase during the first 48 h. As many of the HSP90 client proteins are needed for cell survival and proliferation [49] use of HSP90 inhibitors induces both cell cycle arrest and apoptosis in cancer cells [50]. Similar results have been seen in canine osteosarcoma and mast cell tumor cell lines treated with either ganetespib or STA-1474, inducing growth inhibition that was at least in part mediated by caspase 3/7-dependent apoptosis [51, 52].

Ganetespib, a second generation HSP90 inhibitor, effectively and simultaneously destabilizes HSP90 client proteins in NSCLC cells including receptor tyrosine kinases and canonical JAK/STAT, PI3K/AKT, MAPK and mTOR signaling. Moreover, ganetespib accumulates in tumors relative to normal tissues, with a half-life in the tumor that is 10- to 19-fold longer than in normal tissues or plasma [23]. Our experiments show that the prodrug of ganetespib, STA-1474, down-regulates signal transduction proteins in a dose-dependent manner. For some proteins (AKT, MAPK, STAT3) only the phosphorylated form is decreased but not the total protein. Lack of total STAT3 down-regulation has been reported before with STA-1474 treatment [51], which is an unexpected finding as STAT3 is a HSP90 client protein. This result can be explained when individual protein turnover rate is considered. HSP90 inhibition should lead to the rapid degradation of newly synthesized proteins and those with a longer half-life will show a slower decrease in loss of protein after the HSP90 inhibition [53].

The tyrosine kinase inhibitor toceranib is a multi-targeted kinase inhibitor that effects both tumor cell proliferation and tumor angiogenesis. We sought to determine if common tyrosine kinases which support lung tumor growth could be inhibited by torceranib. Even after 72 h of exposure to the highest dose of torceranib used (1 μM) this compound was unable to abrogate the phosphorylated and total protein expression of all the kinases evaluated in both cell lines. Further studies are needed to evaluate if torceranib demonstrates inhibitory activity against VEGFR, PDGFR and c-Kit protein expression in canine lung cancer cell lines.

Exposure of tumor cells to HSP90 inhibitors induces a cellular protective and compensatory response which is to increase the expression of other heat shock proteins, notably HSP70. The increase of HSP70 expression has been shown to abrogate the extent of cell death [50]. Indeed we saw a reciprocal upregulation of HSP70 when both cell lines were treated with increasing doses of HSP90 inhibitors. Previous studies have reported an increase in HSP70 protein expression when canine tumors and cancer cell lines were treated with STA-1474 [26, 51].

HSP70 has also been shown to be a driver of oncogenesis therefore therapy using a combination of a HSP90 inhibitor with a HSP70 inhibitor may provide a wider therapeutic window and increase the target-driven therapeutic index. We wanted to investigate if the simultaneous inhibition of HSP70 would enhance the growth inhibitory effects of HSP90 treatment. The use of VER155008, a HSP70 inhibitor, suppressed cell growth in both cell lines but only at high μM concentrations. Similar results have been described in human NSCLC cells treated with this drug [54]. Surprisingly when the cell lines were treated with the fixed ratio combinations of STA-1474 and VER155008 based on their ICs_50_, the doublet combination effects were different not only between cell lines but also in terms of the desired effect level. Moderate synergy was seen when the BACA line was treated with the combination at concentrations higher than its’ IC_50_ values, whereas in the CLAC line this combination created synergism only at the IC_50_ value. This was an unexpected finding, as VER155008 has shown to be synergistic with other HSP90 inhibitors in human NSCLC lines [54–56]. Multiple drugs can compete with each other for the same transporter, molecular target or have conflicting effects on the cell cycle. Studies suggest that cancer cells are sensitive to multiple drugs at a certain drug mixing ratio, and that the optimal mixing ratio must be retained in tumor tissues to achieve the maximal drug combination effect [57–59]. The lack of a constant synergistic effect at all levels with the combination therapy may be due to the induction of other protective HSPs or upregulation of alternative oncogenic compensatory pathways not examined in this study.

Evaluation of drug activity using a two-dimensional monolayer of tumor cells poorly models the disease *in vivo*. Tumors are three-dimensional (3D) complex tissues composed of neoplastic cells, vasculature and tumor stroma. As such, we sought to determine the response of STA-1474 in a 3D-model of canine lung cancer as well as in cells of the tumor stroma, represented by tumor-stromal fibroblasts obtained from a primary canine lung tumor cell culture. Both cell lines grew as spheres in ultra-low attachment wells, as described by Vinci *et al*. [60]. In contrast to other studies, the need to add growth factors to the media to foster the formation of spheroids was not needed [61]. This was critical for the interpretation of the ICs_50_ as addition of growth factors can enhance cell proliferation and viability making direct comparison of spheroid and monolayer ICs_50_ impossible. Spheroid conformation was different between the cell lines. This finding was similar to previous reports that have reported different cell lines from a variety of tumors grow in different spheroid patterns [60, 62]. The spheroid shape may influence drug responsiveness. Morphometric analysis of sphere area would be helpful to determine relative cell viability after drug exposure. However, although the sphere area can be measured using image analysis software, determination of area can be ambiguous in approximately 50% of the evaluated cases [62]. The STA-1474 ICs_50_ were higher for both spheroid cell cultures than for the monolayer adherent cultures. This was not unexpected as in general chemotherapy drugs have increased potency in 2D models compared with spheroid cultures, although exceptions do exist with a better response seen in 3D models to some pharmaceuticals [62]. The drug effectiveness in limiting spheroid viability is in part due to the ability of the agent to diffuse into the sphere. Therefore, although spheroids are a better model to evaluate cellular responsiveness to drugs, caution must be used when interpreting the ICs_50_ as lack of vascularization responsible for the delivery and distribution of the agent throughout the sphere is not present.

The tumor stroma is composed of cancer-associated fibroblasts (CAFs), cells that are phenotypically and functionally different from their normal counterparts. CAFs can induce cancer cell stemness as well as epithelial to mesenchymal transition to promote tumor progression [63]. CAFs can also induce therapeutic resistance in NSCLC [64]. Moreover, cytotoxic treatment can increase both CAF percentage and cytokine secretion, especially IL-17A, both of which contribute to cancer-initiating cells growth and therapeutic resistance [65]. However, stromal fibroblasts also respond to the neoplastic epithelial cells by expressing growth factors [66]. Because FGFR3 is a known HSP90 client protein [67] we treated tumor stromal fibroblasts with STA-1474. Although the IC_50_ for tumor stromal fibroblasts was higher than the cell lines it was still within a biologically achievable dose. This result was dissimilar to what has been reported with other HSP90 inhibitors, which have decreased CAFs cytokine production but not viability [68].

## Conclusions

According to our genetic and biochemical analyses, canine lung adenocarcinoma cell lines are relevant to the same human cancer. Likewise, the results of the canine aCGH and the variability in biological response to the chemotherpy agents found in these two cell lines suggests that the canine patient would benefit from precision medicine which has significantly improved the quality of life of the human lung cancer patient. We showed that treatment with STA-1474 decreases BACA and CLAC viability and HSP90 client protein expression at biologically relevant doses, by inducing dose- and time-dependent apoptosis. In contrast with other small molecules inhibitors, STA-1474 affects several proteins in different cellular signaling pathways and decreases viability not only in tumor cells, but also stromal fibroblasts. The efficacy of two small molecule inhibitors in canine lung cancer cell lines grown in a 3D spheroid format established that ICs_50_ will be increased when compared to the ICs_50_ obtained when cells are grown in a monolayer. This finding re-enforces the importance of evaluating cellular responses to drugs in a model that more accurately mimics the natural tumor environment. Nevertheless, the preclinical activity profile of STA-1474 demonstrated in this study provides preliminary evidence that this compound is superior to most of the currently available drugs and may offer an effective therapeutic opportunity to manage the canine lung cancer patient.

## Supporting Information

S1 Table Comparative genomic hybridization analysis. Contains segmentation, classification of focal *vs*. large alterations and gene annotation.
(XLSX)

## Abbreviations

HSP90: heat shock protein ninety
HSP70: heat shock protein seventy
EGFR: epidermal growth factor receptor
c-Kit: v-kit Hardy-Zuckerman 4 feline sarcoma viral oncogene homolog
HER2: human epidermal growth factor receptor 2
VEGFR: vascular endothelial growth factor receptor
PDGFRα/β: platelet-derived growth factor receptor alpha and beta
c-MET: c-mesenchymal epithelial transition factor
MAPK: mitogen-activated protein kinases
c-RET: c-rearranged during transfection
AKT-2: v-akt murine thymoma viral oncogene homolog 2
NKX2-1: NK2 homebox 1
TTF-1: thyroid transcription factor 1
IGF-IRβ: insulin growth factor I
STAT3: signal transducer and activator of transcription 3
GAPDH: glyceraldehyde-3-phosphate dehydrogenase
NDUFA1: NADH dehydrogenase (ubiquinone) 1 alpha subcomplex, 1
ALK: anaplastic lymphoma kinase
FACS: fluorescence-activated cell sorting
CI: combination index
DRI: dose reduction index
FGFR3: fibroblast growth factor receptor 3
CAFs: cancer associated fibroblasts
CGH: comparative genomic hybridization
CNAs: copy number alterations
CaGe: cancer gene annotation system for cancer genomics
